# Milk-Sickness

**Published:** 1854-07

**Authors:** A. G. Henry

**Affiliations:** Lafayette, Oregon


### ART. IV.—MILK-SICKNESS.

BY A. G. HENRY, M. D., IN A LETTER TO THE EDITOR.

*Dear Sir:*

I noticed in your Journal, for June, a paper from Dr. THOMPSON, on milk-sickness, in which he advances the opinion, that the disease is of malarious origin, instead of animal or mineral, and his facts go very far, in my judgment, to confirm that view of the subject. During my residence in Pekin, on the Illinois river, in the years 1845-6 and '7, I investigated the history of the disease in connection with its existence in a particular locality in that vicinity, and the investigation developed the following facts:

The disease had prevailed to a considerable extent in a particular locality on the Mackinaw creek, some two or three miles from where it empties into the Illinois river. The people in the vicinity had located the cause of the disease within the compass of a few hundred yards on the Mackinaw bottom, and its greatest prevalence in the fall, after the grass upon the prairies had dried up. The cattle then took to the bottom, and soon showed symptoms of the disease. This was in October, after the frosts had killed the vegetation on the prairies, and after the malarious influences of that very highly malarious region had ceased to operate upon the inhabitants in the vicinity. The cattle that were kept from this locality, although within reach of the malarious influence, never showed symptoms of the disease. No individual in that vicinity for once questioned its animal origin, and that animals contracted the disease from eating a particular vegetable peculiar to the locality, and which remained green after the grasses had dried up; and the following facts amount to very strong presumptive evidence of the propriety of these conclusions.

The disease had showed itself, for many years, about the same time in the fall, without any exception until the fall of 1844.

That season was a very wet one, and the Illinois river rose to an unusual height, and remained up until late in the season, consequently the back-water from the Illinois, with the high state of Mackinaw, kept this particular locality submerged, for the *first time* for many years, until all the annual vegetables were destroyed, so that when the bottom became dry in September the locality was entirely bare of vegetation.

The usual malarious diseases of that vicinity obtained their usual prevalence, and without any unusual precaution, *no case of milk-sickness, either in man or beast, was known in that vicinity that fall.* The spring following (1845) I removed to Pekin, and learning the foregoing fact, I had my attention particularly directed to the matter. I resided three years in Pekin, and I heard of no case of milk-sickness in that locality until the fall of 1848, when it re-appeared as formerly. During my residence of three years in Pekin, I practiced in the neighborhood where there were more cases of malarious disease, than in any other neighborhood in the vicinity, yet nothing simulating milk-sickness was met with.

The disease in this locality could not have depended upon "*a mineral*," for no change had taken place in the springs or other water-courses, to explain the immunity from the disease for three years. But we have, to my mind, a very satisfactory cause for the suspension of the disease in the killing of the vegetation, and which required some three years for its re-production.

It may be explained very plausibly, perhaps, upon the principle of malarious influence, but the foregoing instance proves to my entire satisfaction that the facts and arguments are altogether in favor of the doctrine of its animal origin through the agency of some unknown vegetable. I have not heard of the existence of the disease on the Pacific coast, where the *Rhus toxicodendron* and every other variety of the plant grows in abundance, and when the ground is covered with snow horses subsist for weeks upon it with entire impunity, while its touch is poisonous to myself and my family.

LAFAYETTE, Oregon, Oct. 2, 1853.

